# Ethyl 1-(4-chloro­benz­yl)-3-phenyl-1*H*-pyrazole-5-carboxyl­ate

**DOI:** 10.1107/S1600536812007428

**Published:** 2012-02-29

**Authors:** Ben-Qian Hao, Wei-Ren Xu, Fan-Cui Meng, Gui-Yun Duan

**Affiliations:** a Taishan Medical College, Tai an 271016, People’s Republic of China; b Tianjin Institute of Pharmaceutical Research, Tian Jin 300193, People’s Republic of China

## Abstract

In the title compound, C_19_H_17_ClN_2_O_2_, the pyrazole ring makes dihedral angles of 6.97 (5) and 79.25 (1)°, respectively, with the phenyl and chlorophenyl rings, respectively. In the crystal, C—H⋯O hydrogen bonds are observed.

## Related literature

For background to the title compound, see: Ge *et al.* (2007 ▶, 2009 ▶, 2011 ▶). For a related compound, see: Xia *et al.* (2007 ▶).
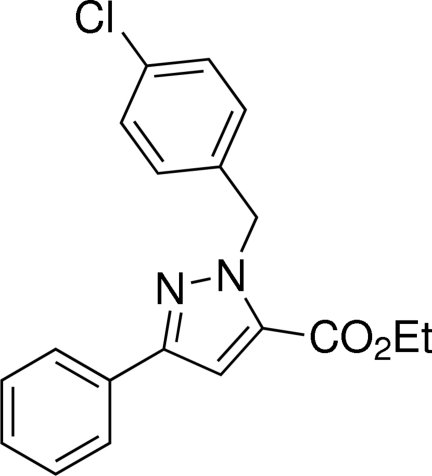


## Experimental

### 

#### Crystal data

C_19_H_17_ClN_2_O_2_
*M* *_r_* = 340.80
Triclinic, 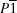

*a* = 8.1815 (10) Å
*b* = 10.4039 (12) Å
*c* = 11.0969 (13) Å
α = 109.981 (2)°
β = 90.107 (2)°
γ = 104.046 (2)°
*V* = 857.43 (18) Å^3^
*Z* = 2
Mo *K*α radiation
μ = 0.24 mm^−1^
*T* = 298 K
0.26 × 0.24 × 0.20 mm

#### Data collection

Bruker SMART CCD diffractometer
Absorption correction: multi-scan (*SADABS*; Sheldrick, 2003 ▶) *T*_min_ = 0.941, *T*_max_ = 0.954
4495 measured reflections
3008 independent reflections
2554 reflections with *I* > 2σ(*I*)
*R*_int_ = 0.023

#### Refinement

*R*[*F* ^2^ > 2σ(*F* ^2^)] = 0.039
*wR*(*F* ^2^) = 0.107
*S* = 1.03
3008 reflections
218 parameters
H-atom parameters constrained
Δρ_max_ = 0.25 e Å^−3^
Δρ_min_ = −0.30 e Å^−3^

### 

Data collection: *SMART* (Bruker, 2005 ▶); cell refinement: *SAINT* (Bruker, 2005 ▶); data reduction: *SAINT*; program(s) used to solve structure: *SHELXS97* (Sheldrick, 2008 ▶); program(s) used to refine structure: *SHELXL97* (Sheldrick, 2008 ▶); molecular graphics: *XP* in *SHELXTL* (Sheldrick, 2008 ▶); software used to prepare material for publication: *SHELXL97*.

## Supplementary Material

Crystal structure: contains datablock(s) I, global. DOI: 10.1107/S1600536812007428/zj2059sup1.cif

Structure factors: contains datablock(s) I. DOI: 10.1107/S1600536812007428/zj2059Isup2.hkl

Supplementary material file. DOI: 10.1107/S1600536812007428/zj2059Isup3.cml

Additional supplementary materials:  crystallographic information; 3D view; checkCIF report

## Figures and Tables

**Table 1:** Hydrogen-bond geometry (Å, °)

*D*—H⋯*A*	*D*—H	H⋯*A*	*D*⋯*A*	*D*—H⋯*A*
C6—H6⋯O1^i^	0.93	2.56	3.281 (2)	135
C1—H1*B*⋯O1	0.97	2.42	2.921 (2)	111

Symmetry code: (i) 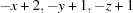.

**Fig. 1.**
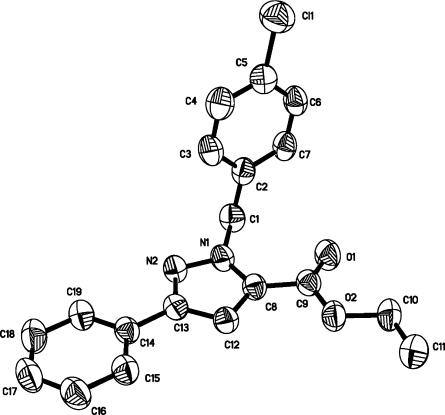
The molecular structure of (I), showing displacement ellipsoids drawn at the 50% probability level.

**Fig. 2.**
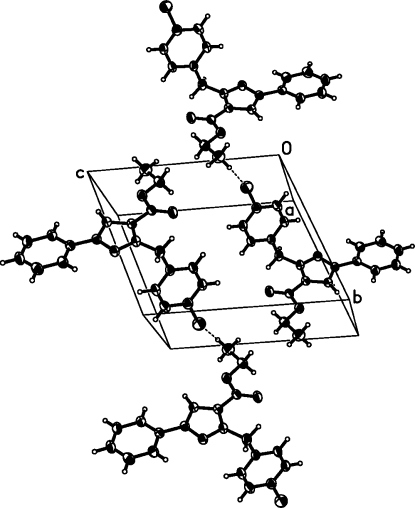
A packing diagram for (I).

## supplementary crystallographic information

### Comment

Synthesis of nitrogen-containing heterocyclic compounds has been a subject of great interest due to the wide application in agrochemical and pharmaceutical fields (Ge *et al.*, 2009, 2011). Some pyrazole derivatives which belong to this category have been of interest for their biological activities. Considerable efforts have been devoted to the development of novel pyrazole compounds. The title pyrazole (I) (Fig. 1) was synthesized in order to study and compare its biological properties with other related compounds (Xia *et al.*, 2007). (I) was screened for anticancer activities and found to be inactive. We report here the crystal structure of the title compound. In the title compound, C_19_H_17_ClN_2_O_2_, all bond lengths and angles show normal values. The pyrazole ring makes dihedral angles of 6.97° and 79.25°, respectively, with the C14–C19 and C2–C7 phenyl rings. There existed intermolecule C—H···O hydrogen bonds to stablized the crystal structure.

### Experimental

A mixture of ethyl 3-phenyl-1*H*-pyrazole-5-carboxylate (0.02 mol), 1-chloro-4-(chloromethyl)benzene (0.0024 mol) and potassium carbonate (0.02 mol) in acetonitrile (100 ml) was heated to reflux for 3 h. The solvent was removed under reduced pressure and a product was isolated by column chromatography on silica gel (yield 82%). Crystals of (I) suitable for X-ray diffraction were obtained by allowing a refluxed solution of the product in ethyl acetate (0.10 *M*) to cool slowly to room temperature (without temperature control) and allowing the solvent to evaporate for 2 d.

### Refinement

All H atoms were placed in geometrically calculated positions and refined using a riding model with C—H = 0.97 Å (for CH_2_ groups) and 0.96 Å (for CH_3_ groups), their isotropic displacement parameters were set to 1.2 times (1.5 times for CH_3_ groups) the equivalent displacement parameter of their parent atoms.

### Crystal data

**Table:**

C_19_H_17_ClN_2_O_2_	*Z* = 2
*M**_r_* = 340.80	*F*(000) = 356
Triclinic, *P*1	*D*_x_ = 1.320 Mg m^−^^3^
*a* = 8.1815 (10) Å	Mo *K*α radiation, λ = 0.71073 Å
*b* = 10.4039 (12) Å	Cell parameters from 2727 reflections
*c* = 11.0969 (13) Å	θ = 2.4–28.2°
α = 109.981 (2)°	µ = 0.24 mm^−^^1^
β = 90.107 (2)°	*T* = 298 K
γ = 104.046 (2)°	Block, white
*V* = 857.43 (18) Å^3^	0.26 × 0.24 × 0.20 mm

### Data collection

**Table:**

Bruker SMART CCD diffractometer	3008 independent reflections
Radiation source: fine-focus sealed tube	2554 reflections with *I* > 2σ(*I*)
Graphite monochromator	*R*_int_ = 0.023
φ and ω scans	θ_max_ = 25.1°, θ_min_ = 2.0°
Absorption correction: multi-scan (*SADABS*; Sheldrick, 2003)	*h* = −9→9
*T*_min_ = 0.941, *T*_max_ = 0.954	*k* = −11→12
4495 measured reflections	*l* = −11→13

### Refinement

**Table:**

Refinement on *F*^2^	Secondary atom site location: difference Fourier map
Least-squares matrix: full	Hydrogen site location: inferred from neighbouring sites
*R*[*F*^2^ > 2σ(*F*^2^)] = 0.039	H-atom parameters constrained
*wR*(*F*^2^) = 0.107	*w* = 1/[σ^2^(*F*_o_^2^) + (0.0481*P*)^2^ + 0.2205*P*] where *P* = (*F*_o_^2^ + 2*F*_c_^2^)/3
*S* = 1.03	(Δ/σ)_max_ < 0.001
3008 reflections	Δρ_max_ = 0.25 e Å^−^^3^
218 parameters	Δρ_min_ = −0.30 e Å^−^^3^
0 restraints	Extinction correction: *SHELXL97* (Sheldrick, 2008), Fc^*^=kFc[1+0.001xFc^2^λ^3^/sin(2θ)]^-1/4^
Primary atom site location: structure-invariant direct methods	Extinction coefficient: 0.082 (5)

### Special details

**Table:**

Geometry. All e.s.d.'s (except the e.s.d. in the dihedral angle between two l.s. planes) are estimated using the full covariance matrix. The cell e.s.d.'s are taken into account individually in the estimation of e.s.d.'s in distances, angles and torsion angles; correlations between e.s.d.'s in cell parameters are only used when they are defined by crystal symmetry. An approximate (isotropic) treatment of cell e.s.d.'s is used for estimating e.s.d.'s involving l.s. planes.
Refinement. Refinement of *F*^2^ against ALL reflections. The weighted *R*-factor *wR* and goodness of fit *S* are based on *F*^2^, conventional *R*-factors *R* are based on *F*, with *F* set to zero for negative *F*^2^. The threshold expression of *F*^2^ > σ(*F*^2^) is used only for calculating *R*-factors(gt) *etc*. and is not relevant to the choice of reflections for refinement. *R*-factors based on *F*^2^ are statistically about twice as large as those based on *F*, and *R*-factors based on ALL data will be even larger.

### Fractional atomic coordinates and isotropic or equivalent isotropic displacement parameters (Å^2^)

**Table:**

	*x*	*y*	*z*	*U*_iso_*/*U*_eq_	
Cl1	1.50815 (7)	0.95990 (7)	0.77388 (6)	0.0839 (2)	
O1	0.84683 (18)	0.38892 (14)	0.64732 (11)	0.0665 (4)	
O2	0.85747 (17)	0.25171 (13)	0.76392 (11)	0.0598 (3)	
N1	0.75907 (17)	0.58814 (15)	0.87794 (13)	0.0492 (3)	
N2	0.71786 (18)	0.65423 (15)	0.99660 (13)	0.0514 (4)	
C1	0.7812 (2)	0.6630 (2)	0.78600 (17)	0.0561 (4)	
H1A	0.7167	0.7334	0.8091	0.067*	
H1B	0.7367	0.5960	0.7006	0.067*	
C2	0.9642 (2)	0.73491 (18)	0.78307 (15)	0.0501 (4)	
C3	1.0607 (3)	0.8300 (2)	0.89533 (17)	0.0650 (5)	
H3	1.0125	0.8475	0.9733	0.078*	
C4	1.2266 (3)	0.8989 (2)	0.89318 (18)	0.0707 (5)	
H4	1.2900	0.9625	0.9689	0.085*	
C5	1.2976 (2)	0.8726 (2)	0.77783 (18)	0.0584 (5)	
C6	1.2062 (2)	0.77923 (19)	0.66533 (16)	0.0563 (4)	
H6	1.2553	0.7623	0.5878	0.068*	
C7	1.0400 (2)	0.71053 (19)	0.66882 (16)	0.0546 (4)	
H7	0.9778	0.6465	0.5928	0.066*	
C8	0.78772 (19)	0.46120 (17)	0.86693 (15)	0.0462 (4)	
C9	0.8332 (2)	0.36663 (18)	0.74715 (15)	0.0491 (4)	
C10	0.9062 (3)	0.1503 (2)	0.65285 (18)	0.0641 (5)	
H10A	1.0026	0.1973	0.6190	0.077*	
H10B	0.8133	0.1060	0.5855	0.077*	
C11	0.9509 (3)	0.0421 (2)	0.6971 (2)	0.0779 (6)	
H11A	1.0399	0.0877	0.7659	0.117*	
H11B	0.9883	−0.0249	0.6267	0.117*	
H11C	0.8533	−0.0063	0.7270	0.117*	
C12	0.7612 (2)	0.44406 (18)	0.98320 (15)	0.0479 (4)	
H12	0.7698	0.3673	1.0054	0.058*	
C13	0.71823 (19)	0.56638 (17)	1.06121 (15)	0.0457 (4)	
C14	0.67851 (19)	0.60557 (18)	1.19642 (15)	0.0473 (4)	
C15	0.6985 (2)	0.5232 (2)	1.26854 (16)	0.0564 (4)	
H15	0.7340	0.4412	1.2303	0.068*	
C16	0.6660 (3)	0.5621 (2)	1.39691 (18)	0.0656 (5)	
H16	0.6798	0.5062	1.4441	0.079*	
C17	0.6135 (3)	0.6829 (2)	1.45453 (18)	0.0676 (5)	
H17	0.5926	0.7095	1.5408	0.081*	
C18	0.5921 (2)	0.7646 (2)	1.38374 (19)	0.0678 (5)	
H18	0.5561	0.8462	1.4226	0.081*	
C19	0.6235 (2)	0.7266 (2)	1.25547 (17)	0.0573 (4)	
H19	0.6077	0.7823	1.2086	0.069*	

### Atomic displacement parameters (Å^2^)

**Table:**

	*U*^11^	*U*^22^	*U*^33^	*U*^12^	*U*^13^	*U*^23^
Cl1	0.0670 (3)	0.0917 (4)	0.0892 (4)	0.0145 (3)	0.0093 (3)	0.0313 (3)
O1	0.0907 (10)	0.0682 (8)	0.0420 (7)	0.0261 (7)	0.0127 (6)	0.0173 (6)
O2	0.0781 (8)	0.0569 (7)	0.0473 (7)	0.0281 (6)	0.0168 (6)	0.0146 (6)
N1	0.0541 (8)	0.0561 (8)	0.0408 (7)	0.0195 (6)	0.0075 (6)	0.0175 (6)
N2	0.0560 (8)	0.0559 (8)	0.0444 (8)	0.0206 (7)	0.0092 (6)	0.0162 (7)
C1	0.0677 (11)	0.0640 (11)	0.0460 (9)	0.0263 (9)	0.0052 (8)	0.0247 (8)
C2	0.0668 (11)	0.0502 (9)	0.0394 (8)	0.0232 (8)	0.0059 (7)	0.0179 (7)
C3	0.0789 (13)	0.0709 (12)	0.0386 (9)	0.0173 (10)	0.0123 (9)	0.0126 (9)
C4	0.0802 (14)	0.0731 (13)	0.0436 (10)	0.0107 (11)	−0.0019 (9)	0.0078 (9)
C5	0.0652 (11)	0.0596 (11)	0.0548 (10)	0.0218 (9)	0.0074 (8)	0.0217 (9)
C6	0.0716 (12)	0.0619 (11)	0.0425 (9)	0.0280 (9)	0.0133 (8)	0.0199 (8)
C7	0.0735 (12)	0.0567 (10)	0.0353 (8)	0.0233 (9)	0.0027 (8)	0.0138 (7)
C8	0.0446 (8)	0.0502 (9)	0.0416 (8)	0.0130 (7)	0.0028 (7)	0.0129 (7)
C9	0.0480 (9)	0.0523 (10)	0.0413 (9)	0.0101 (7)	0.0031 (7)	0.0116 (7)
C10	0.0764 (13)	0.0584 (11)	0.0516 (10)	0.0247 (10)	0.0141 (9)	0.0070 (9)
C11	0.0853 (15)	0.0660 (13)	0.0883 (16)	0.0333 (11)	0.0253 (12)	0.0252 (12)
C12	0.0499 (9)	0.0502 (9)	0.0439 (9)	0.0142 (7)	0.0049 (7)	0.0158 (7)
C13	0.0419 (8)	0.0513 (9)	0.0419 (8)	0.0117 (7)	0.0034 (6)	0.0144 (7)
C14	0.0408 (8)	0.0534 (9)	0.0426 (9)	0.0100 (7)	0.0042 (6)	0.0121 (7)
C15	0.0632 (11)	0.0588 (10)	0.0461 (9)	0.0171 (9)	0.0082 (8)	0.0161 (8)
C16	0.0717 (12)	0.0778 (13)	0.0475 (10)	0.0166 (10)	0.0096 (9)	0.0242 (10)
C17	0.0681 (12)	0.0852 (14)	0.0425 (10)	0.0185 (10)	0.0140 (8)	0.0149 (10)
C18	0.0641 (12)	0.0720 (13)	0.0585 (11)	0.0247 (10)	0.0132 (9)	0.0070 (10)
C19	0.0573 (10)	0.0629 (11)	0.0513 (10)	0.0220 (9)	0.0094 (8)	0.0151 (9)

### Geometric parameters (Å, º)

**Table:**

Cl1—C5	1.750 (2)	C8—C9	1.469 (2)
O1—C9	1.207 (2)	C10—C11	1.490 (3)
O2—C9	1.331 (2)	C10—H10A	0.9700
O2—C10	1.452 (2)	C10—H10B	0.9700
N1—N2	1.3475 (19)	C11—H11A	0.9600
N1—C8	1.362 (2)	C11—H11B	0.9600
N1—C1	1.468 (2)	C11—H11C	0.9600
N2—C13	1.341 (2)	C12—C13	1.399 (2)
C1—C2	1.508 (3)	C12—H12	0.9300
C1—H1A	0.9700	C13—C14	1.473 (2)
C1—H1B	0.9700	C14—C19	1.389 (2)
C2—C7	1.384 (2)	C14—C15	1.392 (2)
C2—C3	1.389 (3)	C15—C16	1.387 (2)
C3—C4	1.377 (3)	C15—H15	0.9300
C3—H3	0.9300	C16—C17	1.374 (3)
C4—C5	1.375 (3)	C16—H16	0.9300
C4—H4	0.9300	C17—C18	1.377 (3)
C5—C6	1.370 (3)	C17—H17	0.9300
C6—C7	1.381 (3)	C18—C19	1.384 (3)
C6—H6	0.9300	C18—H18	0.9300
C7—H7	0.9300	C19—H19	0.9300
C8—C12	1.372 (2)		
			
C9—O2—C10	115.81 (13)	O2—C10—H10A	110.3
N2—N1—C8	111.51 (13)	C11—C10—H10A	110.3
N2—N1—C1	118.60 (14)	O2—C10—H10B	110.3
C8—N1—C1	129.64 (14)	C11—C10—H10B	110.3
C13—N2—N1	105.49 (13)	H10A—C10—H10B	108.6
N1—C1—C2	112.37 (13)	C10—C11—H11A	109.5
N1—C1—H1A	109.1	C10—C11—H11B	109.5
C2—C1—H1A	109.1	H11A—C11—H11B	109.5
N1—C1—H1B	109.1	C10—C11—H11C	109.5
C2—C1—H1B	109.1	H11A—C11—H11C	109.5
H1A—C1—H1B	107.9	H11B—C11—H11C	109.5
C7—C2—C3	118.03 (17)	C8—C12—C13	105.44 (15)
C7—C2—C1	121.30 (16)	C8—C12—H12	127.3
C3—C2—C1	120.66 (15)	C13—C12—H12	127.3
C4—C3—C2	121.10 (17)	N2—C13—C12	110.70 (14)
C4—C3—H3	119.5	N2—C13—C14	120.36 (14)
C2—C3—H3	119.5	C12—C13—C14	128.93 (15)
C5—C4—C3	119.29 (18)	C19—C14—C15	118.53 (16)
C5—C4—H4	120.4	C19—C14—C13	120.92 (15)
C3—C4—H4	120.4	C15—C14—C13	120.54 (15)
C6—C5—C4	121.16 (18)	C16—C15—C14	120.64 (17)
C6—C5—Cl1	119.15 (14)	C16—C15—H15	119.7
C4—C5—Cl1	119.69 (15)	C14—C15—H15	119.7
C5—C6—C7	118.99 (16)	C17—C16—C15	120.21 (19)
C5—C6—H6	120.5	C17—C16—H16	119.9
C7—C6—H6	120.5	C15—C16—H16	119.9
C6—C7—C2	121.43 (16)	C16—C17—C18	119.58 (18)
C6—C7—H7	119.3	C16—C17—H17	120.2
C2—C7—H7	119.3	C18—C17—H17	120.2
N1—C8—C12	106.85 (14)	C17—C18—C19	120.77 (18)
N1—C8—C9	122.81 (14)	C17—C18—H18	119.6
C12—C8—C9	130.33 (15)	C19—C18—H18	119.6
O1—C9—O2	124.39 (15)	C18—C19—C14	120.25 (18)
O1—C9—C8	125.32 (16)	C18—C19—H19	119.9
O2—C9—C8	110.29 (14)	C14—C19—H19	119.9
O2—C10—C11	107.06 (16)		
			
C8—N1—N2—C13	−0.84 (18)	C12—C8—C9—O1	176.62 (18)
C1—N1—N2—C13	−175.68 (14)	N1—C8—C9—O2	178.64 (14)
N2—N1—C1—C2	96.41 (17)	C12—C8—C9—O2	−3.3 (2)
C8—N1—C1—C2	−77.4 (2)	C9—O2—C10—C11	171.93 (16)
N1—C1—C2—C7	126.30 (17)	N1—C8—C12—C13	−0.64 (17)
N1—C1—C2—C3	−55.0 (2)	C9—C8—C12—C13	−178.92 (16)
C7—C2—C3—C4	0.4 (3)	N1—N2—C13—C12	0.41 (18)
C1—C2—C3—C4	−178.33 (18)	N1—N2—C13—C14	179.81 (13)
C2—C3—C4—C5	−0.1 (3)	C8—C12—C13—N2	0.15 (18)
C3—C4—C5—C6	−0.1 (3)	C8—C12—C13—C14	−179.18 (15)
C3—C4—C5—Cl1	179.85 (16)	N2—C13—C14—C19	6.6 (2)
C4—C5—C6—C7	−0.1 (3)	C12—C13—C14—C19	−174.15 (17)
Cl1—C5—C6—C7	179.98 (13)	N2—C13—C14—C15	−172.21 (15)
C5—C6—C7—C2	0.5 (3)	C12—C13—C14—C15	7.1 (3)
C3—C2—C7—C6	−0.6 (3)	C19—C14—C15—C16	−0.7 (3)
C1—C2—C7—C6	178.15 (15)	C13—C14—C15—C16	178.08 (16)
N2—N1—C8—C12	0.95 (18)	C14—C15—C16—C17	0.0 (3)
C1—N1—C8—C12	175.06 (16)	C15—C16—C17—C18	0.5 (3)
N2—N1—C8—C9	179.39 (14)	C16—C17—C18—C19	−0.3 (3)
C1—N1—C8—C9	−6.5 (3)	C17—C18—C19—C14	−0.5 (3)
C10—O2—C9—O1	1.1 (3)	C15—C14—C19—C18	1.0 (3)
C10—O2—C9—C8	−178.97 (14)	C13—C14—C19—C18	−177.83 (16)
N1—C8—C9—O1	−1.4 (3)		

### Hydrogen-bond geometry (Å, º)

**Table:**

*D*—H···*A*	*D*—H	H···*A*	*D*···*A*	*D*—H···*A*
C6—H6···O1^i^	0.93	2.56	3.281 (2)	135
C1—H1*B*···O1	0.97	2.42	2.921 (2)	111

Symmetry code: (i) −*x*+2, −*y*+1, −*z*+1.
